# Predictors and outcomes of recurrent retroperitoneal liposarcoma with multiple tumors

**DOI:** 10.3389/fmed.2023.1161494

**Published:** 2023-09-08

**Authors:** Huan Deng, Xingming Xu, Jingwang Gao, Jun Huang, Guibin Liu, Liqiang Song, Bo Wei

**Affiliations:** ^1^Department of Gastrointestinal Surgery, Peking University First Hospital, Beijing, China; ^2^Department of General Surgery, The First Medical Center, Chinese People’s Liberation Army General Hospital, Beijing, China

**Keywords:** retroperitoneal liposarcoma, recurrent, multiple tumors, pathological differentiation, local recurrence, predictor, nomogram model

## Abstract

**Background:**

Retroperitoneal liposarcoma (RLS) is a rare but severe disease. Repeated postoperative recurrence with multiple tumors is a therapeutic dilemma. The clinical outcomes and survival predictors of recurrent RLS with multiple tumors remain to be explored.

**Methods:**

Patients with recurrent RLS were retrospectively analyzed. Univariate and multivariate analysis was performed to find independent prognostic factors that were correlated with Overall survival (OS) or progression-free survival (PFS). Factors significant in univariate analysis were further included into multivariate Cox proportional hazards regression model. The nomogram model was built to predict the survival status of patients. Variables that were significant in multivariable analysis were added to the internally validated nomogram models. The analysis of OS and PFS was performed by Kaplan–Meier analysis and log-rank test.

**Results:**

A total of 113 recurrent RLS patients with multiple tumors were enrolled in the study. The 1-, 3-, and 5-years OS (PFS) rates were 70.7% (76.1%), 35.9% (76.1%), and 30.9% (76.1%), respectively. Univariate and multivariate analyses showed that number of surgeries, resection methods, tumor size, status of pathological differentiation, pathological subtypes, and recurrence patterns were important prognostic factors for OS or PFS (each *p* < 0.05). Nomogram models were established to efficiently predict the prognostic status of patients. Patients with the local recurrence (LR) pattern had a poor prognosis and would derive no survival benefit from combined organ resection and R0/R1 resection (each *p* < 0.05).

**Conclusion:**

RLS patients recurrence with multiple tumors had a poor prognosis. Those patients should be followed up more frequently after surgery. The strategies of aggressive resection may not improve the survival of patients with LR pattern in the retroperitoneum. Prognostic factors in the efficient nomogram models should be considered in the individualized clinical management of recurrent RLS with multiple tumors.

## Introduction

Retroperitoneal liposarcoma (RLS) is the most common type of retroperitoneal sarcoma (1). According to official classifications, RLS can be further divided into the following four subtypes: well-differentiated liposarcoma (WDL), dedifferentiated liposarcoma (DDL), myxoid cell liposarcoma (MLS), and pleomorphic liposarcoma (PLS) (2, 3). Due to the tumor growth in the deep abdomen, RLS without typical symptoms, evading the early clinical detection. A large number of patients with RLS tend to have the possibility of recurrence. The prognosis of patients with recurrent RLS is not optimistic, although great improvements in treatment have been achieved in recent years. Postoperative recurrence with multiple tumors is a frequent outcome among patients with RLS. Usually, those patients with multiple tumors has a poor prognosis (4). Therefore, there is an urgent need to find high-efficient predictive factors for the prognosis of recurrent RLS with multiple tumors.

Numerous studies have investigated the clinicopathological features and outcomes of recurrent RLS. Some studies have demonstrated that radical resection of tumors will improve the survival status of patients with RLS (5). However, the multiple and large volume of tumors in recurrent RLS may restrict the ability of surgeon to achieve a true radical resection (6). The complicated anatomic structures of recurrent RLS also limit the potential of the complete surgical margin, which associated with a poor prognosis. Likewise, complete capsular resection and combined organ resection are difficlut to achieve during the surgical procedures of recurrent RLS (7). These factors are therapeutic obstacle and poor predictors for patients with recurrent RLS.

The pathological subtype is an important prognostic factor for the survival of patients with recurrent RLS (8). During clinical practice, we found that pathological subtype could alter as the tumor number increasing in some recurrent cases. The phenomenon indicates that tumor number may associated with the status of pathological differentiation. However, no related studies have clarified the potential mechanism of this correlation.

The prognosis of recurrent RLS also correlated with the relapsed tumor numbers. Previous studies have demonstrated that RLS patients usually growth with multiple tumors simultaneously (4). Although radical resection is currently the effective treatment for RLS, recurrent cases with multiple tumors has higher propensity of postoperative recurrence and poor prognosis (9). The tumor numbers have been proved as a poor prognostic factor for RLS. Nevertheless, research on the characteristics and prognostic outcomes of recurrent RLS with multiple tumors is currently limited. Clarification the effects of tumor numbers on the prognosis of recurrent RLS would be helpful for the better understanding of recurrence mechanism and providing clinical management strategies.

We analyzed the basic clinical and pathological features of recurrent RLS with multiple tumors to investigate the important prognostic predictors and outcomes in the subset of patients. This study may provide evidence for individualized clinical management strategies for recurrent RLS with multiple tumors.

## Materials and methods

### Patient selection

All patients with RLS experienced multiple recurrences and underwent at least two surgeries from February 2000 to August 2017 at the First Medical Center, Chinese People Liberation Army General Hospital. The pathological subtype was diagnosed and confirmed by experienced pathologists based on WHO (World Health Organization) pathologic criteria (10, 11). Patients with distant organ metastasis were excluded in the study. Patients with adjuvant radiotherapy or chemotherapy at any point of treatment were also excluded. Patients who experienced recurrence but without complete medical records or follow-up data were excluded. The recurrence confirmation of RLS combined preoperative radiological examinations, postoperative pathological examinations and surgical records. All patients signed the consent forms for the series of studies. This study was approved by the Protection of Human Subjects Committee of the Chinese People’s Liberation Army General Hospital.

### Definitions

Multiple tumors were defined as the presence of two or more non-contiguous multifocal RLS concurrently. Local recurrence (LR) was defined as tumor relapse at the same anatomical compartment in the retroperitoneum. Non-LR was defined as tumor recurrence at another compartment but without distant organ metastasis (4). Tumor growth rate (TGR) was defined as tumor size (the maximum dimension of the largest mass recorded on final pathological records) divided by the time from last resection to this recurrence diagnosed (12). Overall survival (OS) referred to the time from surgical resection to the end of 5-years follow-up or death. Progression-free survival (PFS) was defined as the time from surgical resection to the initial data of documented tumor progression or death within 5-years (13).

### Statistical analyses

Continuous variables are expressed as the median (Q1–Q3). The categorical data are expressed as frequencies (percentages), it compared using the chi-square or Fisher exact test. Univariate and multivariate analyses were applied for the exploration of independent prognostic factors that were associated with OS or PFS. Factors significant in univariate analysis were further included into multivariate Cox proportional hazards regression model. The Cox regression models were conducted by the survival coxph function of the R package. The nomogram models based on Cox regression were built to predict the survival status of recurrent RLS patients with multiple tumors. The Kaplan–Meier curves were applied to estimate the OS and PFS. Data were analyzed using IBM SPSS Statistics (Version 25.0). A two-sided *p* value <0.05 was considered statistically significant.

## Results

### Basic clinicopathological characteristics

**Figure 1:**
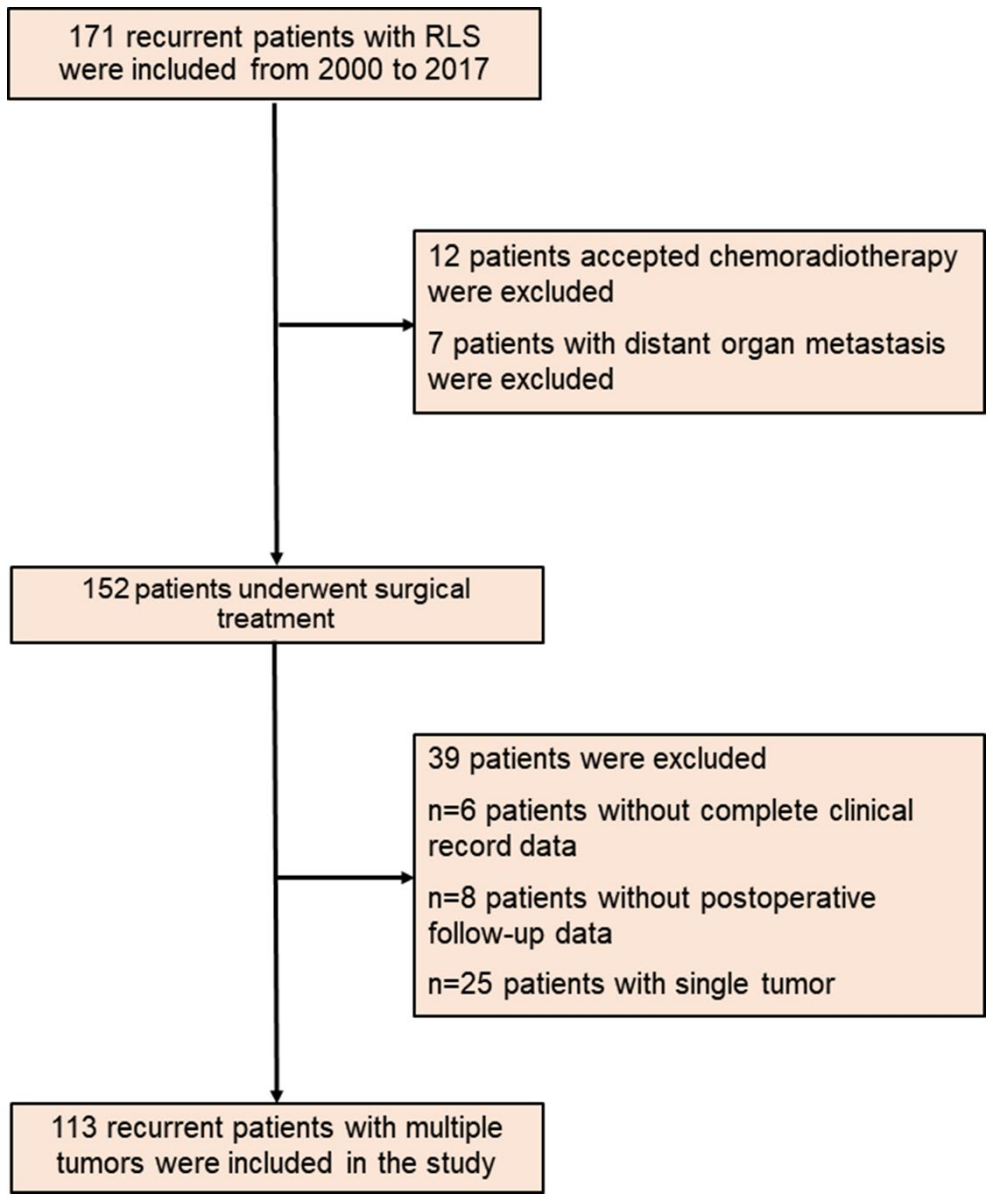
The flow diagram of recurrent RLS patients with multiple tumors. RLS, retroperitoneal liposarcoma.

Based on the above enrollment criteria, one hundred thirteen recurrent RLS patients with multiple tumors were finally enrolled (Figure 1). Sixty-two patients were male, fifty-one patients were female, and the median age of all patients was 53-years. The median tumor size was 18 cm, and the median TGR was 1.29 cm/month. Fifty-four percent of patients had a change of pathological differentiation, and almost half of patients experienced recurrence at the local sites in the retroperitoneum. The important clinical and pathological characteristics of patients with multiple tumors are shown in Table 1.

**Table 1:** Demographic, clinical, and pathological characteristics of included patients with recurrent RLS.

Characteristics	Value
Age at surgery (years)	53 (44–61)
Gender	
Male	62 (54.9%)
Female	51 (45.1%)
ASA score (points)	2 (2–3)
The number of surgeries	3 (2–4)
Tumor growth rate (cm/month)	1.29 (0.62–2.86)
Invaded vessel	
No	61 (54.0%)
Yes	52 (46.0%)
Resection methods	
R0 (Negative gross margin)	46 (40.7%)
R1 (Positive gross margin)	45 (39.8%)
R2 (Palliative resection)	22 (19.5%)
Tumor size (cm)	18 (13–25)
Completeness of tumor capsule	
Complete	62 (54.9%)
Incomplete	51 (45.1%)
Status of pathological differentiation	
Consistent	52 (46.0%)
Change	61 (54.0%)
Combined organ resection	
No	38 (33.6%)
Yes	75 (66.4%)
Recurrence patterns	
Non-LR	57 (50.4%)
LR	56 (49.6%)
Pathological subtypes	
WDL	34 (30.1%)
MLS	30 (26.6%)
PLS	17 (15.0%)
DDL	32 (28.3%)
Pathological classification	
WDL	34 (30.1%)
Non-WDL	79 (69.9%)

RLS, retroperitoneal liposarcoma; LR, local recurrence; WDL, well-differentiated liposarcoma; DDL, dedifferentiated liposarcoma; MLS, myxoid cell liposarcoma; PLS, pleomorphic liposarcoma; ASA, American society of anesthesiologists.

### Predictors for OS and PFS

Univariate and multivariate analyses were performed to explore clinicopathologic variables associated with 5-years OS (Table 2). The results showed that resection method, tumor size, status of pathological differentiation, and recurrence pattern were independent risk factors for OS (each *p* < 0.05). Likewise, we analyzed the prognostic factors associated with 5-years PFS for all patients. The univariate and multivariate analyses showed that number of surgeries, resection method, recurrence pattern and pathological subtype were the significant factors associated with PFS (each *p* < 0.05) (Table 3). Considering the importance of the recurrence pattern of recurrent RLS, we further divided the cohort into two groups. The comparison of clinicopathological feature between LR and non-LR patterns showed that pathological subtypes and status of pathological differentiation were significantly different (each *p* < 0.05) (Table 4).

**Table 2:** Univariate and multivariate analysis of clinicopathologic variables associated with 5-years OS.

Characteristics	Total (*N*)	Univariate analysis		Multivariate analysis	
		HR (95% CI)	*p* value	HR (95% CI)	*p* value
Age at surgery (years)	113				
≤53	59	Reference			
>53	54	1.570 (0.985–2.500)	0.058	1.406 (0.855–2.311)	0.179
Gender	113				
Male	62	Reference			
Female	51	1.326 (0.834–2.108)	0.233		
ASA score (points)	113				
≤2	84	Reference			
>2	29	1.577 (0.938–2.651)	0.086	1.256 (0.699–2.256)	0.445
The number of surgeries	113				
2–3	82	Reference			
>3	31	1.319 (0.793–2.194)	0.287		
Tumor growth rate (cm/month)	113				
≤1.29	56	Reference			
>1.29	57	1.573 (0.988–2.506)	0.056	1.156 (0.687–1.946)	0.585
Invaded vessel	113				
No	61	Reference			
Yes	52	1.236 (0.778–1.963)	0.370		
Resection methods	113				
R0 (Negative gross margin)	46	Reference			
R1 (Positive gross margin)	45	2.024 (1.170–3.499)	**0.012**	1.982 (1.104–3.559)	**0.022**
R2 (Palliative resection)	22	3.971 (2.139–7.370)	**<0.001**	3.404 (1.762–6.575)	**<0.001**
Tumor size (cm)	113				
≤18	58	Reference			
>18	55	1.848 (1.158–2.948)	**0.010**	1.670 (1.004–2.778)	**0.048**
Completeness of tumor capsule	113				
Complete	62	Reference			
Incomplete	51	1.240 (0.778–1.977)	0.365		
Status of pathological differentiation	113				
Consistent	52	Reference			
Change	61	1.670 (1.042–2.677)	**0.033**	1.929 (1.147–3.244)	**0.013**
Combined organ resection	113				
No	38	Reference			
Yes	75	0.951 (0.585–1.546)	0.840		
Recurrence patterns	113				
Non-LR	57	Reference			
LR	56	1.806 (1.129–2.890)	**0.014**	1.995 (1.193–3.338)	**0.008**
Pathological classification	113				
WDL	34	Reference			
Non-WDL	79	1.626 (0.953–2.776)	0.074	1.568 (0.895–2.745)	0.116

LR, local recurrence; OS, overall survival; RLS, retroperitoneal liposarcoma; HR, hazard ratio; CI, confidence interval; ASA, American society of anesthesiologists. The cutoff value was the median value of variable. Bold *p* value refers to *p* < 0.05.

**Table 3:** Univariate and multivariate analysis of clinicopathologic variables associated with 5-years PFS.

Characteristics	Total (*N*)	Univariate analysis		Multivariate analysis	
		HR (95% CI)	*p* value	HR (95% CI)	*p* value
Age at surgery (years)	113				
≤53	59	Reference			
>53	54	1.304 (0.872–1.950)	0.196		
Gender	113				
Male	62	Reference			
Female	51	0.963 (0.642–1.443)	0.854		
ASA score (points)	113				
≤2	84	Reference			
>2	29	1.995 (1.256–3.168)	**0.003**	1.347 (0.805–2.253)	0.256
The number of surgeries	113				
2–3	82	Reference			
>3	31	1.469 (0.938–2.303)	0.093	1.626 (1.014–2.606)	**0.043**
Tumor growth rate (cm/month)	113				
≤1.29	56	Reference			
>1.29	57	1.702 (1.130–2.564)	**0.011**	1.567 (0.989–2.483)	0.056
Invaded vessel	113				
No	61	Reference			
Yes	52	1.213 (0.810–1.816)	0.349		
Resection methods	113				
R0 (Negative gross margin)	46	Reference			
R1 (Positive gross margin)	45	1.415 (0.875–2.289)	0.157	1.599 (0.958–2.671)	0.073
R2 (Palliative resection)	22	3.393 (1.932–5.959)	**<0.001**	3.181 (1.728–5.855)	**<0.001**
Tumor size (cm)	113				
≤18	58	Reference			
>18	55	1.451 (0.969–2.173)	0.071	1.167 (0.761–1.790)	0.479
Completeness of Tumor capsule	113				
Complete	62	Reference			
Incomplete	51	1.064 (0.703–1.612)	0.769		
Status of pathological differentiation	113				
Consistent	52	Reference			
Change	61	0.986 (0.661–1.473)	0.947		
Combined organ resection	113				
No	38	Reference			
Yes	75	1.150 (0.743–1.779)	0.531		
Recurrence patterns	113				
Non-LR	57	Reference			
LR	56	1.666 (1.107–2.507)	**0.014**	2.021 (1.309–3.120)	**0.002**
Pathological classification	113				
WDL	34	Reference			
Non-WDL	79	1.700 (1.080–2.675)	**0.022**	1.705 (1.057–2.752)	**0.029**

LR, local recurrence; RFS, recurrence-free survival; RLS, retroperitoneal liposarcoma; HR, hazard ratio; CI, confidence interval; ASA, American society of anesthesiologists. The cutoff value was the median value of variable. Bold *p* value refers to *p* < 0.05.

**Table 4:** Clinicopathological features comparison between non-LR and LR patterns in all patients.

Clinicopathological features	Non-LR (*n* = 57)	LR (*n* = 56)	*p* value
Age at surgery (years)			0.302
≤53	33 (57.9%)	26 (46.4%)	
>53	24 (42.1%)	30 (53.6%)	
Gender			0.110
Male	21 (36.8%)	30 (53.6%)	
Female	36 (63.2%)	26 (46.4%)	
ASA score			0.707
≤2	41 (71.9%)	43 (76.8%)	
>2	16 (28.1%)	13 (23.2%)	
The number of surgeries			0.103
2–3	37 (64.9%)	45 (80.4%)	
>3	20 (35.1%)	11 (19.6%)	
Tumor growth rate (cm/month)			1.000
≤1.29	28 (49.1%)	28 (50%)	
>1.29	29 (50.9%)	28 (50%)	
Invaded vessel			0.074
Yes	36 (63.2%)	25 (44.6%)	
No	21 (36.8%)	31 (55.4%)	
Resection methods			0.467
R0 (Negative gross margin)	26 (45.6%)	20 (35.7%)	
R1 (Positive gross margin)	22 (38.6%)	23 (41.1%)	
R2 (Palliative resection)	9 (15.8%)	13 (23.2%)	
Tumor size (cm)			0.927
≤18	30 (52.6%)	28 (50%)	
>18	27 (47.4%)	28 (50%)	
Completeness of tumor capsule			0.110
Complete	36 (63.2%)	26 (46.4%)	
Incomplete	21 (36.8%)	30 (53.6%)	
Pathological subtypes			**0.038**
WDL	15 (26.3%)	17 (30.4%)	
MLS	14 (24.6%)	16 (28.6%)	
PLS	14 (24.6%)	3 (5.4%)	
DDL	14 (24.6%)	20 (35.7%)	
Pathological classification			0.277
WDL	43 (75.4%)	36 (64.3%)	
Non-WDL	14 (24.6%)	20 (35.7%)	
Status of pathological differentiation			**< 0.001**
Change	17 (29.8%)	35 (62.5%)	
Consistent	40 (70.2%)	21 (37.5%)	
Combined organ resection			0.596
Yes	21 (36.8%)	17 (30.4%)	
NO	36 (63.2%)	39 (69.6%)	

LR, local recurrence; RLS, retroperitoneal liposarcoma; WDL, well-differentiated liposarcoma; DDL, dedifferentiated liposarcoma; MLS, myxoid cell liposarcoma; PLS, pleomorphic liposarcoma; ASA, American society of anesthesiologists. The cutoff value was the median value of variable. Bold *p* value refers to *p* < 0.05.

### Nomogram models to predict prognostic status

**Figure 2:**
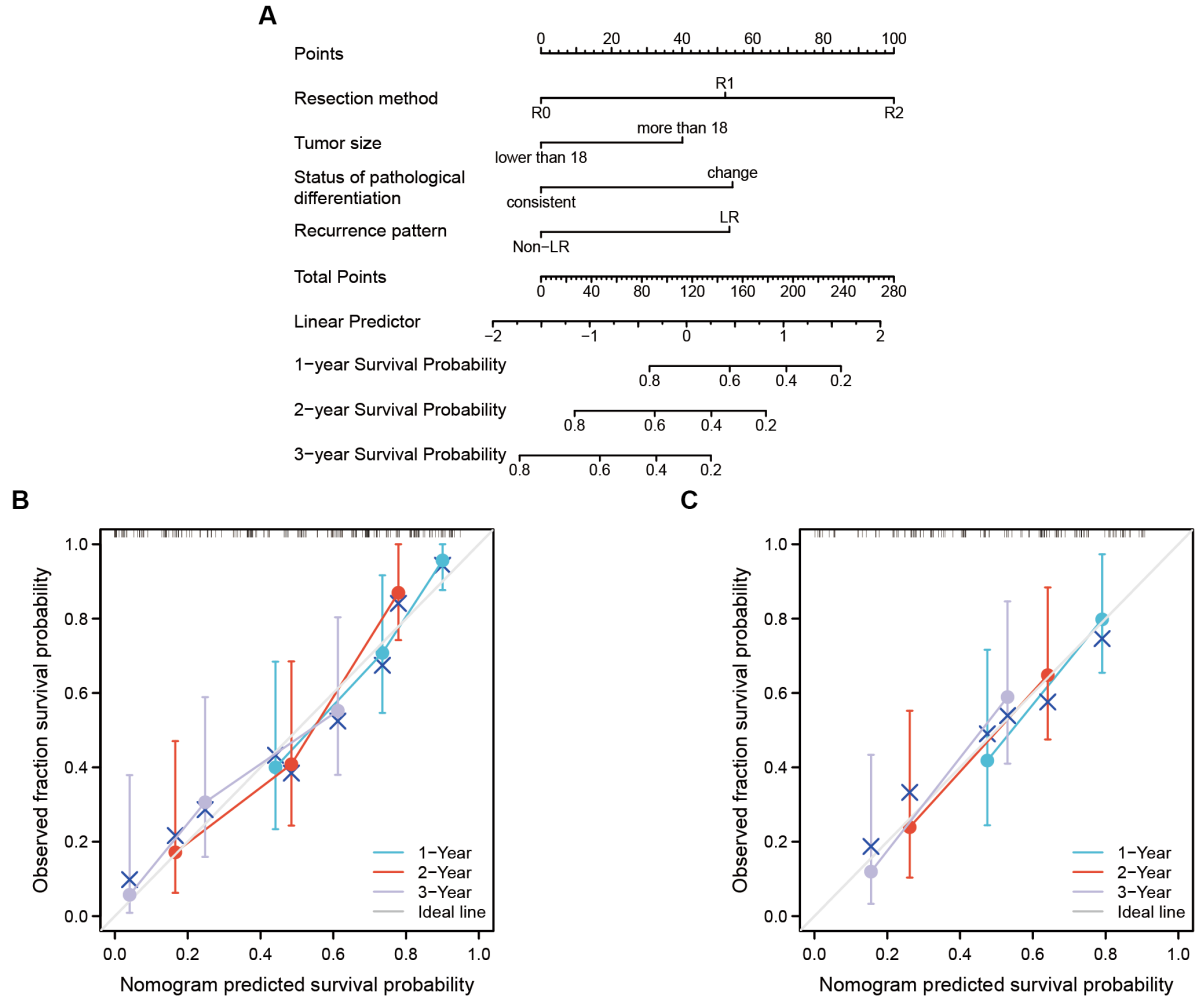
The nomogram model was built to predict the OS of recurrent RLS patients with multiple tumors. **(A)** Nomogram for 1-year, 2-years and 3-years overall survival in all enrolled patients. **(B)** Calibration plots of training set for 1-year, 2-years, and 3-years OS in all enrolled patients. **(C)** Calibration plots of validation set for 1-year, 2-years, and 3-years OS in all enrolled patients. The X-axis: bootstrap-predicted survival; the Y-axis: actual outcome. LR, local recurrence; RLS, retroperitoneal liposarcoma; OS, overall survival. The cutoff value of tumor size was the median value of the variable (the median value included in the lower side; the 5-years OS was too low to be displayed in the nomogram model).

The predictors enrolled in nomogram models were significant factors in the multivariate Cox model. The models can be applied to accurately predict the OS rate of RLS patients who experienced recurrence with multiple tumors (Figure 2). Each predictor corresponds to a point on the top of the nomogram model. We added each score of the four risk factors to obtain a total score, which indicated the overall risk of survival in a patient. The three horizontal lines on the bottom of the nomogram corresponds to the predicted 1-, 2-, and 3-years OS. A vertical line that would intersect the total point axis and the 1-, 2-, and 3-years survival axis indicate the probability of 1-, 2-, and 3-years of OS predicted by the model (Figure 2A). We further built the internal validated models by dividing the cases into training (60%) and test sets (40%). The calibration curves showed an optimal agreement between the predicted and observed OS. The concordance index for the nomogram was 0.703 (95% CI 0.623–0.783) in the training set (Figure 2B) and 0.695 (95% CI 0.565–0.825) in the test set (Figure 2C).

**Figure 3:**
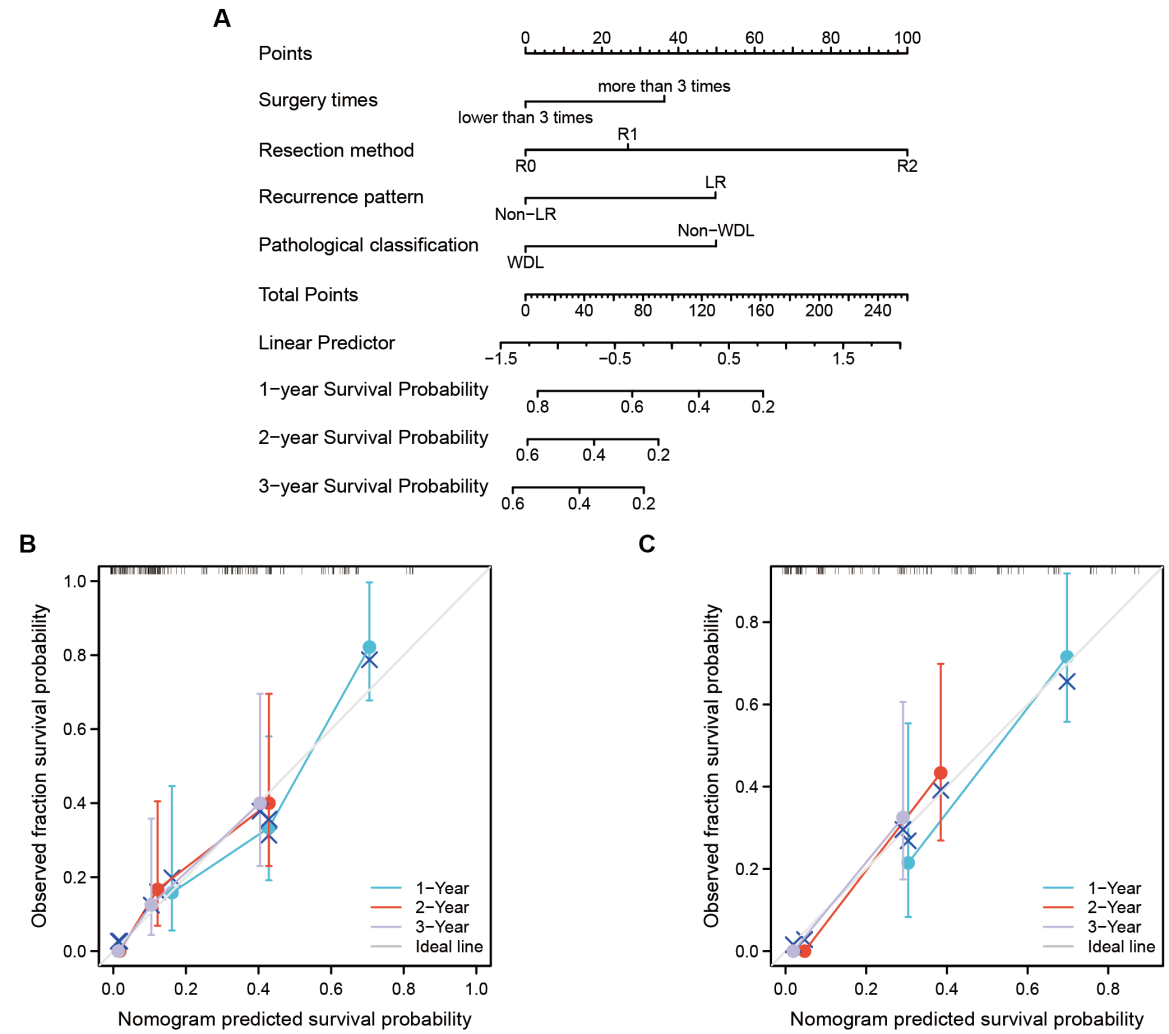
The nomogram model was built to predict the PFS of recurrent RLS patients with multiple tumors. **(A)** Nomogram for 1-year, 2-years, and 3-years PFS in all enrolled patients. **(B)** Calibration plots of training set for 1-year, 2-years, and 3-years PFS in all enrolled patients. **(C)** Calibration plots of validation set for 1-year, 2-years, and 3-years PFS in all enrolled patients. The X-axis: bootstrap-predicted survival; the Y-axis: actual outcome. LR, local recurrence; RLS, retroperitoneal liposarcoma; PFS, progression-free survival. The cutoff value of surgery times was the median value of the variable (the median value included in the lower side; the 5-years PFS was too low to be displayed in the nomogram model).

**Figure 4:**
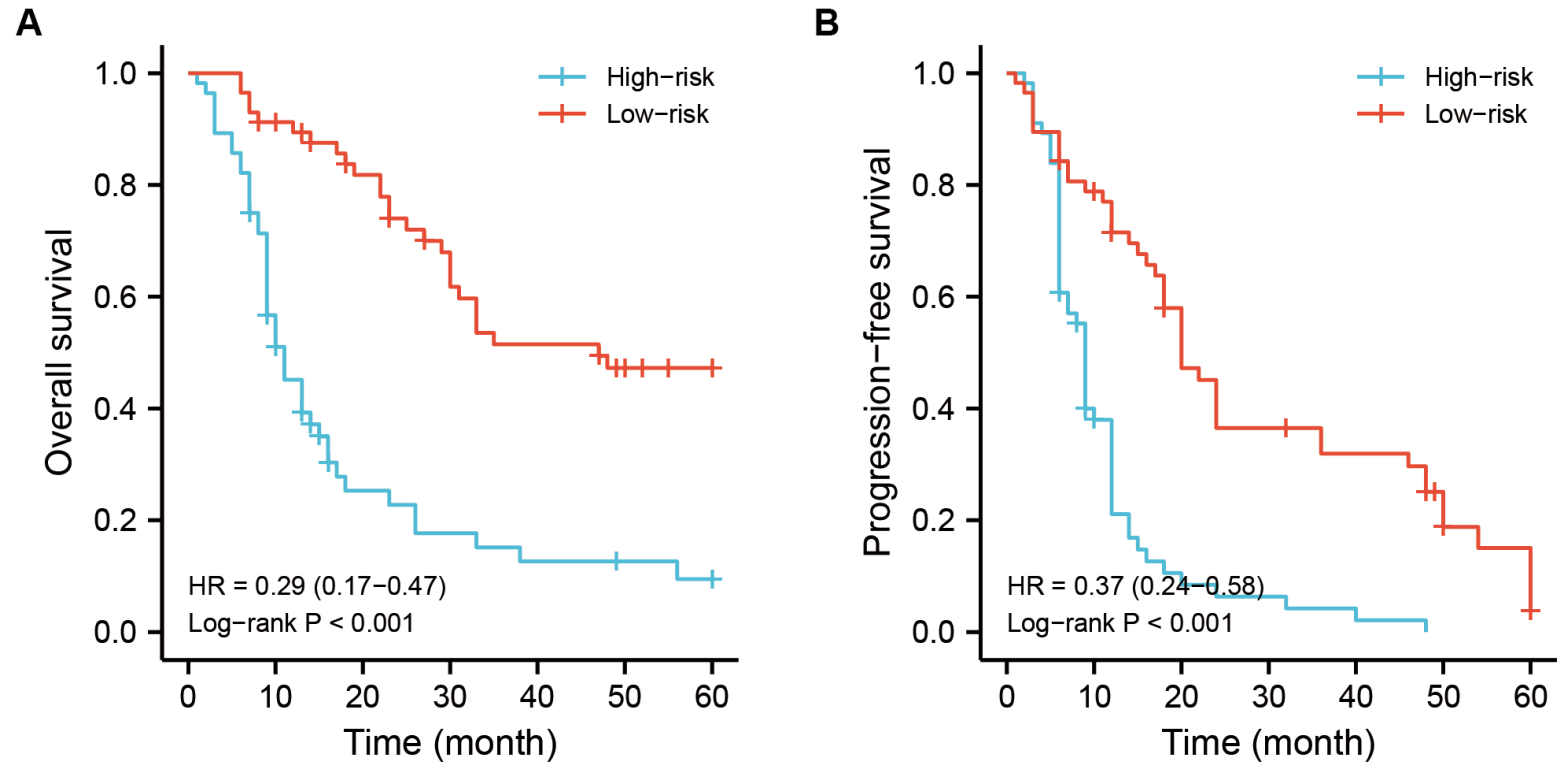
The prognostic analysis of recurrent RLS patients with multiple tumors based on risk scores predicted by nomogram models. **(A)** The OS analysis of enrolled patients in different risk grade. **(B)** The PFS analysis of enrolled patients in different risk grade. The cutoff value of risk score was the median value of the risk scores in all patients. RLS, retroperitoneal liposarcoma; LR, local recurrence; OS, overall survival.

Another nomogram model was established to predict 1-, 2-, and 3-years PFS (Figure 3A). The internal validation again demonstrated an optimal agreement between the predicted and observed PFS. The concordance index for the nomogram was 0.703 (95% CI 0.623–0.783) in the training set (Figure 3B) and 0.695 (95% CI 0.565–0.825) in the test set (Figure 3C). Based on the nomogram models, the cohort was divided into two groups according to the overall risk score. The Kaplan–Meier analyses showed a significant difference in 5-years survival status between the low-risk group and the high-risk group (Figure 4).

### Overall survival and progression-free survival

**Figure 5:**
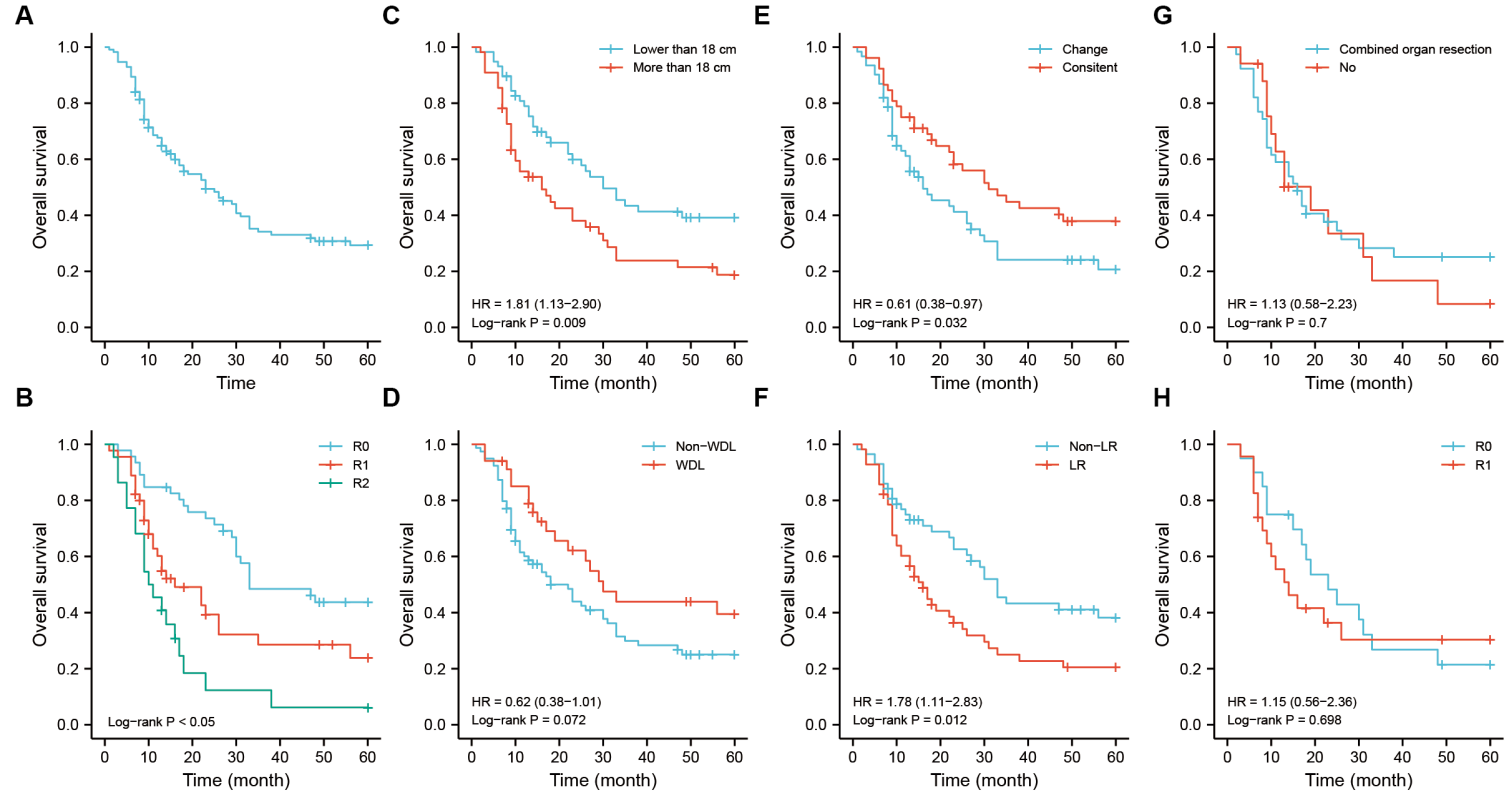
The OS analysis of recurrent RLS patients with multiple tumors. **(A)** The OS analysis of 113 recurrent RLS patients with multiple tumors. **(B)** The OS analysis of 113 enrolled patients with three resection methods. **(C)** The OS analysis of enrolled patients with two types of tumor size. **(D)** The OS analysis of 113 enrolled patients with two types of pathological classification. **(E)** The OS analysis of 113 enrolled patients with different status of pathological differentiation. **(F)** The OS analysis of 113 enrolled patients with different recurrence patterns. **(G)** The OS analysis of enrolled patients with LR pattern after combined organ resection. **(H)** The OS analysis of enrolled patients with LR pattern after R0/R1 resection. The cutoff value of tumor size was the median value of the variable (the median value included in the lower side). OS, overall survival; RLS, retroperitoneal liposarcoma; LR, local recurrence.

The Kaplan–Meier analysis showed that the median survival time of patients was approximately 23-months in this study. The 1-, 3-, and 5-years OS rates were 70.7, 35.9, and 30.9%, respectively (Figure 5A). The main causes of death in the study was showed in the Supplementary Table S1. The resection method had an important prognostic value for OS. Patients with R0 resection had a better prognosis in the entire cohort (Figure 5B). Tumor size lower than 18 cm was associated with a better OS (Figure 5C). The difference of survival outcome was not significant between the WDL and non-WDL pathological subtypes (Figure 5D). However, patients had a better prognosis if the pathological subtype was consistent with that of the last occurrence (Figure 5E). Recurrence pattern is an important predictor for prognosis. In this study, we found that patients who experienced recurrence with the LR pattern had poor OS (Figure 5F). For patients with the LR pattern, the strategy of combined organ resection seemed to have no effect on OS (Figure 5G). Similarly, patients with the LR pattern did not obtain a survival benefit from the R0 or R1 resection method (Figure 5H).

**Figure 6:**
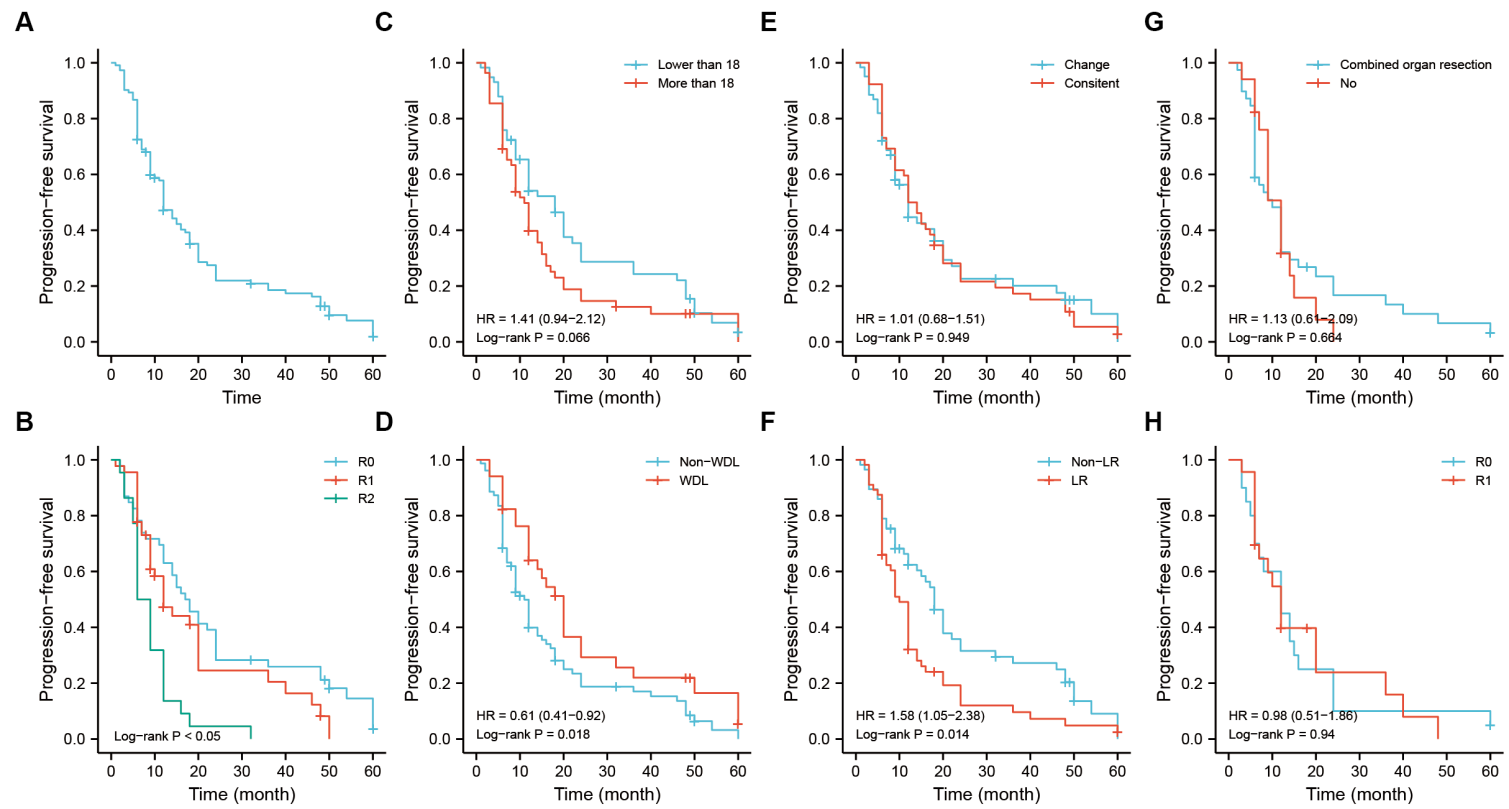
The PFS analysis of recurrent RLS patients with multiple tumors. **(A)** The PFS analysis of 113 recurrent RLS patients with multiple tumors. **(B)** The PFS analysis of 113 enrolled patients with three resection methods. **(C)** The PFS analysis of enrolled patients with two types of tumor size. **(D)** The PFS analysis of 113 enrolled patients with two types of pathological classification. **(E)** The PFS analysis of 113 enrolled patients with different status of pathological differentiation. **(F)** The PFS analysis of 113 enrolled patients with different recurrence patterns. **(G)** The PFS analysis of enrolled patients with LR pattern after combined organ resection. **(H)** The PFS analysis of enrolled patients with LR pattern after R0/R1 resection. The cutoff value of tumor size was the median value of the variable (the median value included in the lower side). PFS, progression-free survival; RLS, retroperitoneal liposarcoma; LR, local recurrence.

The Kaplan–Meier analysis illustrated that the median progression time of patients was approximately 23-months. The 1-, 3-, and 5-years PFS rates were 76.1, 50.8, and 34.4%, respectively (Figure 6A). Patients with R0 and R1 resection had better PFS than those with R2 resection (Figure 6B). The PFS was not significant among the tumor size-stratified groups in the study (Figure 6C). However, patients with the WDL subtype had better PFS regardless of the status of pathological differentiation (Figures 6D,E). As in the OS analysis, patients with the LR pattern exhibited poor PFS, and strategies of combined organ resection and R0/R1 resection did not contribute the survival to those patients (Figures 6F,H).

## Discussion

RLS is a potentially severe disease, need a deeper understanding of the disease mechanisms. WDL and DDL are the most common RLS subtypes representing 40–45% of all RLSs (3). Patients with RLS are susceptible to relapses and even death, has a poor prognosis (14, 15). Recurrent RLS with multiple tumors is a more malignant and aggressive liposarcoma, has poor clinical outcomes (16). The main limiting factor for the long-term survival of RLS patients with multiple tumors is repeated recurrences regardless of the treatment method (17, 18). Hence, it is necessary to clarify factors related to recurrence with multiple tumors and explore the predictors of survival status.

Previous studies have explored the risk factors influencing the prognosis of RLS (19, 20). In this study, we investigated the clinicopathological features of recurrent RLS with multiple tumors and explored the surgical factors that correlated with OS or PFS. The univariate and multivariate analyses showed that number of surgeries, tumor size, pathological subtype, status of pathological differentiation, resection method and recurrence pattern were important prognostic factors of OS and PFS. Nomogram models based on multivariate analysis have been built to predict the survival status of RLS (2, 21). However, a highly efficient model that predicting the prognosis of recurrent RLS with multiple tumors is lacking. Gronchi et al. built a high-efficient model to predict the OS of patients with retroperitoneal soft tissue sarcoma. They enrolled the FNCLCC grade, histologic subtype, extent of resection, multifocality, and tumor size into the models. Interestingly, they found the patients with multifocal disease had better OS in their cohort, the new finding deserves praise and spread (22). Our internal validated models demonstrated an optimal agreement between the predicted and observed OS and PFS. In addition, the risk score–stratified survival analysis revealed the validity of the models. In previous studies, the recurrence pattern was rarely included in the nomogram models (2, 20, 23). We found that the recurrence with LR pattern were the poor prognostic factor in our nomogram models. Consequently, the above prognostic factors input into the nomogram models could be considered in the individualized clinical management of recurrent RLS with multiple tumors.

The number of surgeries and tumor size are important predictors for the prognosis of recurrent RLS. We found that the number of surgeries was an independent risk factor for PFS, and patients who underwent more than three surgeries had a poor prognosis (Table 3). Ishii et al. concluded that recurrence rate might increase as the frequency of recurrence and surgery increased in RLS (18). Tumor size has been seen as an important predictor of recurrent RLS (24). The giant tumor volume of RLS limits the ability of surgeon to achieve the radical resection of tumors. In this study, we found that patients with tumor sizes greater than 18 cm behaved poor OS, which is consistent with the previous study (12).

The pathological subtype of RLS plays an important role in the survival prediction. A number of studies have proved that patients with well differentiated RLS benefited better survival than those with lower differentiation (8, 25). However, this feature is a little different from recurrent patients with multiple tumors. Our survival analysis showed that patients with the WDL subtype had better PFS but without a significant difference in OS. This phenomenon may result from the change of pathological differentiation during recurrence. During clinical practice, we found that some cases repeated recurrence with multiple tumors. It induces that the recurrence cases could not maintain the original status of pathological differentiation. Surprisingly, a large number of recurrence cases grew with multiple tumors, which underwent a change of pathological differentiation (Table 1). In the study finished by Tseng et al. (4), the proportion of multifocal disease in recurrent RLS accounting for approximately 50%. The phenomenon mainly correlated with multiple potential tumor growth point in the retroperitoneum cavity. Some previous studies found that different pathological subtypes of RLS has specific typical gene aberrations and biological features, and the multiple gene aberrations can exist in a patient simultaneously (26–28). It is possible that the tumor growth point with a new pathological subtype conceals in the distant retroperitoneum cavity, exhibiting a change of pathological differentiation during recurrence. It inevitably impacts the survial status of those patients. The uncertainty of pathological differentiation during recurrence influences the long-term survival outcomes of patients. Therefore, patients with the WDL subtype could exhibit better PFS in once recurrence, but the long-term OS may not be benefited. The research conducted by Carolyn et al. also found that the changes of pathological differentiation could impact the prognosis of RLS (29). In our study, patients with the change of pathological differentiation behaved poor OS, whereas, the PFS was not influenced regardless of the status of pathological differentiation. We believe that patients of RLS recurrence with an uncertain pathology, which might influence the long-term OS. However, recurrent patients with consistent pathological differentiation benefited better long-term prognosis (Figure 5E). In the study, RLS patients recurrence with multifocal tumors were more likely to experience the change of pathological differentiation, which resulting the relatively poor prognosis.

The postoperative recurrence is a frequent event in RLS. Local recurrence influences the prognosis of patients with recurrent RLS. However, there is no reliable consensus for the definition of the range of local recurrence in RLS. Some studies defined all recurrences in the retroperitoneum or intra-abdominal regions as local recurrences (5, 17, 29). Distant recurrence has been deemed as tumor recurrence at another compartment of the retroperitoneum or distant organ metastases (4, 30). To explore the outcomes of local recurrence in recurrent RLS, we defined an explicit anatomical ranges of local recurrence in the retroperitoneum. The LR pattern of RLS was defined as the liposarcoma repeated recurrence in the same compartment of the retroperitoneum. The further survival analysis illustrated that patients with the LR pattern had poor OS and PFS. Tseng et al. reported that the differences of OS between patients with multifocal and unifocal disease were dependent on the disease status. They found the patients with multifocal disease or recurrent status had poor prognosis. It is the first literature that described the unusual pattern of multifocal recurrence in RLS (4).

In previous studies, complete resection and combined organ resection have been recommended as the most effective treatments for the recurrence of RLS (9, 31, 32). In our entire cohort, the patients with multiple tumors, who underwent the R0 resection had a better prognosis than those with R1/R2 resection. We further conducted a subgroup survival analysis on patients with the LR pattern. The results showed that those patients with the LR pattern behaved poor OS and PFS. The strategies of combined organ resection and R0/R1 resection would not contribute to the survival of patients, who experienced local recurrence with multiple tumors. These observations indicate that comprehensive strategies need to be considered when applying the aggressive resection to those patients. The follow up strategy should be re-consider for RLS patients. In the previous researches and protocols, the follow up interval was recommended every 6 or 12-months. However, patients with multiple tumors had shorter recurrence intervals. Combining research findings, we suggest shorter interval of follow up for RLS patients with multiple tumors.

### Study limitations

Our study has several limitations. First, this study involved only a single institution, the number of cases was limited, and adjuvant therapies and distant metastases were unable to be further analyzed. Second, the long-term survival and disease-specific death for recurrent RLS should be evaluated. Third, our cases were retrotspective, mainly based on our institution’s medical records, and lacked prospective data. Fourth, the tumor necrosis and the mitotic count that used in the FNCLCC grading, were missed in this retrospective study, it induced the effective sarculator system could not used in the study.

## Conclusion

Prognostic factors that significant in the nomogram models could be considered into the individualized clinical management of recurrent RLS. Those patients recurrence with multiple tumors had a poor prognosis, and should be followed up more frequently after surgery. The strategies of aggressive resection may not improve the survival of patients, who experienced local recurrence with multiple tumors in the retroperitoneum.

## Data availability statement

The datasets presented in this article are not readily available because institution limitations. Requests to access the datasets should be directed to BW, weibo@vip.163.com.

## Ethics statement

This study was approved by the Protection of Human Subjects Committee of the Chinese People’s Liberation Army General Hospital. All patients signed consent forms for the study.

## Author contributions

HD: conceptualisation, data curation, investigation, project administration, writing-original draft, and writing and editing. JG and XX: data curation, investigation, methodology, and formal analysis. GL: built the calibration plots of the dynamic OS nomogram. LS: writing abstract and editing the writing-original draft. JH: data analysis and table editing. BW: conceptualisation, research investigation, project administration, and editing-original draft. All authors contributed to the article and approved the submitted version.

## Funding

This project was supported by grants from the National Natural Science Foundation of China (81773135) and National Key Research and Development Project of China (2019YFB1311505).

## Conflict of interest

The authors declare that the research was conducted in the absence of any commercial or financial relationships that could be construed as a potential conflict of interest.

## Publisher’s note

All claims expressed in this article are solely those of the authors and do not necessarily represent those of their affiliated organizations, or those of the publisher, the editors and the reviewers. Any product that may be evaluated in this article, or claim that may be made by its manufacturer, is not guaranteed or endorsed by the publisher.

## Supplementary material

The Supplementary material for this article can be found online at: https://www.frontiersin.org/articles/10.3389/fmed.2023.1161494/full#supplementary-material
